# Psychometric properties of the online Satisfaction with Life Scale in university students from a low-income region

**DOI:** 10.1186/s41155-023-00254-2

**Published:** 2023-05-17

**Authors:** Antonio Gibran de Almeida Cardoso, Millena Vaz de Carvalho, Maria Isabela Alves de Almeida Silva, Alaiana Marinho Franco, Fernando Rodrigues Peixoto Quaresma, Erika Da Silva Maciel, Marcus Vinicius Nascimento-Ferreira

**Affiliations:** 1 Health, Physical Activity and Behavior Research (HEALTHY-BRA) Group, Universidade Federal Do Tocantins, Miracema Do Tocantins, Brazil; 2 Instituto de Ensino Superior Do Sul Do Maranhão (IESMA/UNISULMA), Imperatriz, Brazil; 3 Programa de Pós-Graduação Em Ensino Em Ciências E Saúde (PPGECS/UFT), Universidade Federal Do Tocantins, Palmas, Tocantins Brazil; 4 Child Cardiovascular Risk and Environmental (YCARE) Research Group, School of Medicine, School of Medicine, University of São Paulo, YouthSão Paulo, Brazil; 5 Universidade Federal Do Tocantins, Campus Universitário de Miracema, Avenida Lourdes Solino S/N°, Setor Universitário, Miracema Do Tocantins, Tocantins 77650000 Brazil

**Keywords:** Questionnaire, Satisfaction with life, Reproducibility, Structural validity, Adults

## Abstract

**Purpose:**

To test the feasibility, reliability, and validity of the Satisfaction with Life Scale (SWLS) in an online format in university students from a low-income region.

**Methods:**

This was a psychometric study, involving a study of reliability (*n* = 117) and validity (*n* = 195) in university students from a region with a Gini index of 0.56. The scale was applied at two time points with an interval of 2 weeks. This scale measures satisfaction with life based on five statements and responses ranging from 1 to 7 (strongly disagree to strongly agree). We conducted the reliability assessment using temporal stability and internal consistency and construct validity assessment by internal structure solution.

**Results:**

All SWLS items showed acceptable (rho > 0.30) and significant (*p* < 0.05) temporal stability and acceptable internal consistency (alpha > 0.70). In construct validity (internal structure), we identified a factor with an explained variance of 59.0% in the exploratory factor analysis. Additionally, in the confirmatory factor analysis, we identified a one-factor structure solution for SWLS with an acceptable model fitting (chi-square/degrees of freedom [*X*^2^/df] = 6.53; Tucker–Lewis Index [TLI] = 0.991; Comparative Fit Index [CFI] = 0.996; root mean square error of approximation [RMSEA] = 0.040; standardized root mean-squared residual [SRMR] = 0.026).

**Conclusion:**

The Satisfaction with Life Scale, in the online format, is a reliable and valid tool for university students in a low-income context.

## Introduction

Satisfaction with life is the subjective method of judging one’s well-being through a broad set of factors related to an individual’s perception of his or her life (Gouveia et al., 2009). The literature has shown that life satisfaction is an important cross-national risk factor for health outcomes in university students (Arrindell et al., 2022; Jensen et al., 2021; Rogowska et al., 2021). Thus, educational institutions may be an ideal setting for raising awareness of mental health issues among undergraduate students (Jensen et al., 2021). In recent years, studies have associated the COVID-19 pandemic with cardiometabolic (Halpern et al., 2021) and mental (Ornell et al., 2020) health issues and with a deterioration of healthy behaviors (Alomari et al., 2020). Additionally, a negative impact of COVID-19 on university students’ life satisfaction, well-being, physical health, physical activity, and some demographic risk factors (e.g., gender, place of residence) was also evidenced in different countries (Rogowska et al., 2021). In this line, special public health attention should be focused on psychologically supporting and monitoring students who have a low level of life satisfaction (Rogowska et al., 2021).

Thus, monitoring satisfaction with life is an important step for maintaining the health of university students, especially in a pandemic (Rogowska et al., 2021). However, measuring this construct poses a challenge (Silva et al., 2021) because satisfaction with life is related to multifactorial characteristics, such as cultural aspects, economic conditions, biological sex, and age (Arrindell et al., 2022; Rogowska et al., 2021). In recent decades, scientists have worked on the construction of valid and comparable tools in different populations. During this period, the Satisfaction with Life Scale (SWLS) was developed to assess global life satisfaction (based on a one-factor structure solution) and does not tap related constructs such as positive affect or loneliness (Diener et al., 1985). The SWLS (based on 5 items with a 7-point Likert style response scale) has become the most widely used measure for assessing life satisfaction (Arrindell et al., 2022) and has been translated and validated into 40 different languages (available at https://eddiener.com/scales/7, accessed on January 31, 2023), including Brazilian Portuguese (Gouveia et al., 2009; Silva et al., 2018, 2021). The SWLS has also received attention from scientists due its low economic and logistical costs, as well as its ability for assessing satisfaction with life in healthy (non-patient) participants in cross-national surveys and also in special populations (Arrindell et al., 2022).

The Brazilian version of the SWLS using a paper-and-pencil self-administered approach was validated in young adults from high-income regions and also showed a one-factor structure solution (Silva et al., 2018, 2021). There is no evidence regarding the structure validity of the SWLS in Brazilian university students from low-income regions. In addition, the literature is scarce on the psychometric properties of the online format of this scale. Although the COVID-19 pandemic has led to an explosion of research (De Man et al., 2021) with a number of tools migrating to the online environment, literacy and Internet access can strongly distort participation and responsiveness to *online* subjective tools, especially in low- and middle-income countries or in societies with large differences in educational and socio-economic levels (De Man et al., 2021; Hensen et al., 2021). To the best of our knowledge, the psychometric properties in the online format were evaluated only in European university students (Rogowska et al., 2021), even then only temporal stability and internal consistency were assessed. Therefore, we aimed to examine the feasibility, reliability, and validity of the SWLS in an online format in university students from a low-income region.

## Methods

### Study design

This was a psychometric study designed to assess the feasibility (adherence to online research), reliability (temporal stability and internal consistency), and construct validity (internal structure) of the online SWLS (Szklo & Nieto, 2014). The study belongs to a multicenter observational longitudinal project entitled 24-h Movement Behavior and Metabolic Syndrome (24 h-MESYN) and was conducted in the 2021 academic year. Detailed information about the 24 h-MESYN study can be found elsewhere (Nascimento-Ferreira et al., 2022a, 2022b).

### Ethical aspects

The study was approved by the Human Research Ethics Committee, opinion number 4.055.604. The study complies with the ethical principles contained in the (I) Declaration of Helsinki, revised in 2008, Seoul, Korea; (II) resolution of CNS 466/12; (III) guidelines for conducting research during the pandemic caused by COVID-19 (available at www.fo.usp.br/wp-content/umails/2020/07/Orientations-conduction-depes research- and activities-CEP.pdf); and (IV) guidelines for research in a virtual environment (CIRCULAR OFÍCIO No. 2/2021/CONEP/SECNS/MS). The study participants were introduced to the study and invited to confirm their participation through an informed consent form. Prior to participation in the study, we informed the students about the data collection methodology and answered any questions.

### Population and sample

The study population consisted of Brazilian university students (*N* = 2225) from a higher education institution selected by convenience in a city located in the south of Maranhão state (Brazil), which has a Gini index of 0.56 (Brasil, 2010). At the end of 2020, the institution informed 2225 students enrolled in nine undergraduate programs: administration, law, physical education, nursing, aesthetics and cosmetics, physiotherapy, nutrition, psychology, and social work, of the study.

We estimated the sample according to the assumptions of Nascimento-Ferreira (Nascimento-Ferreira et al., 2018a, 2018b). To calculate the sample size, we adopted the following parameters: *α* of 0.05, *β* of 0.10 (or power of 90%), and correlation coefficient of 0.30, as the minimum necessary for a correlation matrix in a study of exploratory factor analysis (Martınez-Gonzalez et al., 2014). Based on these specifications, we estimated a sample of 85 university students for the current study. Anticipating losses and rejections of 50% (Morgado et al., 2017), in the test-rest assessment, we established a minimum sample of 170 students for the study of the psychometric properties of the SWLS*.* However, this phase of the 24 h-MESYN project was designed to test the psychometric properties of another five subjective tools (Nascimento-Ferreira et al., 2022a, 2022b) such as the *International Physical Activity Questionnaire* (Nascimento-Ferreira et al., 2022a, 2022b) and the *Dutch Eating Behavior Questionnaire* (Carvalho et al., 2023), for example. Thus, according to the sample parameters of the SWLS and the aforementioned tools, we established inviting 342 students to achieve an acceptable level of statistical power (e.g., sample power of 80.0%) for the psychometric assessments for all tested tools (Nascimento-Ferreira et al., 2022a, 2022b). Next, we conducted a stratified random sampling (Szklo & Nieto, 2014) based on previous cohorts (Barbosa et al., 2016; Cena et al., 2021) with the following strata: sex (at least 60.0% for females), age (at least 25.0% for students up to 20 years of age), and study program (at least 60.0% in the health area) in order to achieve sample diversity. All potential participants were addressed in the university entrance hall and open areas following health recommendations.

### Inclusion and exclusion criteria

We included students enrolled in the institution who were at least 18 years of age and who signed the informed consent form. We excluded from this study students who did not complete the SWLS.

### Study variables

We evaluated the variables sex, age, nature of the course of study, study shift, and satisfaction with life.

### Instruments

We evaluated satisfaction with life via the SWLS (Gouveia et al., 2009; Silva et al., 2018, 2021). We adopted the Brazilian Portuguese version, which was translated by Gouveia et al. (2009) from the original scale (Diener et al., 1985). This version was validated for use in Brazilian young adults (Silva et al., 2018, 2021), with acceptable reliability (internal consistency; Cronbach’s alpha = 0.87) (Silva et al., 2021) and validity (structure with one-factor solution) (Silva et al., 2018, 2021). In the latter psychometric property, the single structural solution in both confirmatory and exploratory factor analysis confirmed the theory and explained > 57% of the total variance of the SWLS construct. The Brazilian version of the SWLS is structured with five statements: (1) “In most respects, my life is close to my ideal”; (2) “The conditions of my life are excellent”; (3) “I am satisfied with my life”; (4) “As much as possible, I have the important things I want in life”; (5) “If I could live a second time, I would change almost nothing in my life” (Gouveia et al., 2009). The responses were measured on a 7-point Likert scale (converted to score) from “strongly disagree” (1 point) to “strongly agree” (7 points); the closer to seven, the greater the satisfaction with life (Gouveia et al., 2009).

Additionally, we subjectively retrieved information about sex (male/female), years (18 to 99), nature of the academic course of study (nursing, physical therapy, nutrition, physical education, aesthetics and cosmetics, psychology, social work, administration, law), and shift (morning, afternoon, night, full). All information was self-reported and collected using an online questionnaire (available at https://forms.gle/L92wXsVaxxfPNgpE8).

### Procedures

The multidisciplinary fieldwork team consisted of undergraduate and graduate researchers in health programs. Previously, the researchers participated in a training program with 20 h of field work for standardization of data collection, training about how to explain the project, initial invitation, and remote contacts with the students. During this training, we also conducted a questionnaire review (Nascimento-Ferreira et al., 2022a, 2022b). The training was offered and supervised at the institution where the data were collected by scientists experienced in this type of study (Nascimento-Ferreira et al., 2018a, 2018b).

We carried out the data collection in three stages. Mandatorily, the first contact (in person) with the participants occurred in the institution’s facilities and in the presence of graduate supervisors*.* At this stage, we explained the study and invited the students to participate, observing all sanitary and institutional standards related to the pandemic. We also sent the link to access the informed consent form via an instant messaging application (WhatsApp). In the second stage, after the electronic signature on the consent form was obtained, the students answered the online questionnaire (first application, Q1). In the third stage, with a 2-week interval, we resent the link, and the participants answered the same questionnaire again (second application, Q2). Here, the questionnaire was sent only to those who replied to it in Q1.

### Data analysis

Statistical analyses were performed using Stata software, version 15.0 (*Stata Corporation, College Station*, TX, USA). For all hypothesis tests, we established a statistical significance level of 95% (*p* ≤ 0.05). The normality of the variables was assessed using the Shapiro–Wilk test. The categorical variables are presented as absolute and relative frequencies, while the continuous variables are described as medians and interquartile ranges. We used the chi-square goodness of fit test to test sample sensitivity (differential bias) (Martınez-Gonzalez et al., 2014).

To test the reliability, we adopted the Spearman correlation coefficient for assessing temporal (test–retest) stability with a cutoff ≥ 0.30 (for acceptable temporal reliability) (Strong et al., 2005) once our data had violated the assumption of normal distribution. In addition, we adopted Cronbach’s coefficient alpha for assessing internal consistency with a cutoff ≥ 0.70 (for acceptable internal reliability) (Fayers & Machin, 2016). To test the validity, we conducted an exploratory factor analysis (to extract the factor structure of our dataset) and a confirmatory factor analysis (to test if the one-factor structure solution [SWLS theoretical model] fits in our dataset). In the exploratory analysis, initially, we conducted the Kaiser–Meyer–Olkin test (KMO > 0.50) and the Bartlett test (*p* < 0.05) to verify whether the data could be factored (Martınez-Gonzalez et al., 2014). Next, we used Varimax and Eigenvalue rotation greater than one for factor extraction (Martınez-Gonzalez et al., 2014). In the confirmatory analysis, we conducted a factor analysis via structural equation modeling with a maximum likelihood estimator (Martınez-Gonzalez et al., 2014). We inserted the measured items (item 1 to item 5) as endogenous variables and the latent construct (satisfaction with life) with an exogenous variable (Martınez-Gonzalez et al., 2014). A graphical representation of the model was obtained using standardized coefficients. The parameters to evaluate the quality of the model were chi-square/degrees of freedom (*X*^2^/df, *p* > 0.05), Tucker–Lewis Index (TLI > 0.95), Comparative Fit Index (CFI > 0.95), root mean square error of approximation (RMSEA < 0.08), and standardized root mean-squared residual (SRMR < 0.08) (Hu & Bentler, 1999).

## Results

**Fig. 1:**
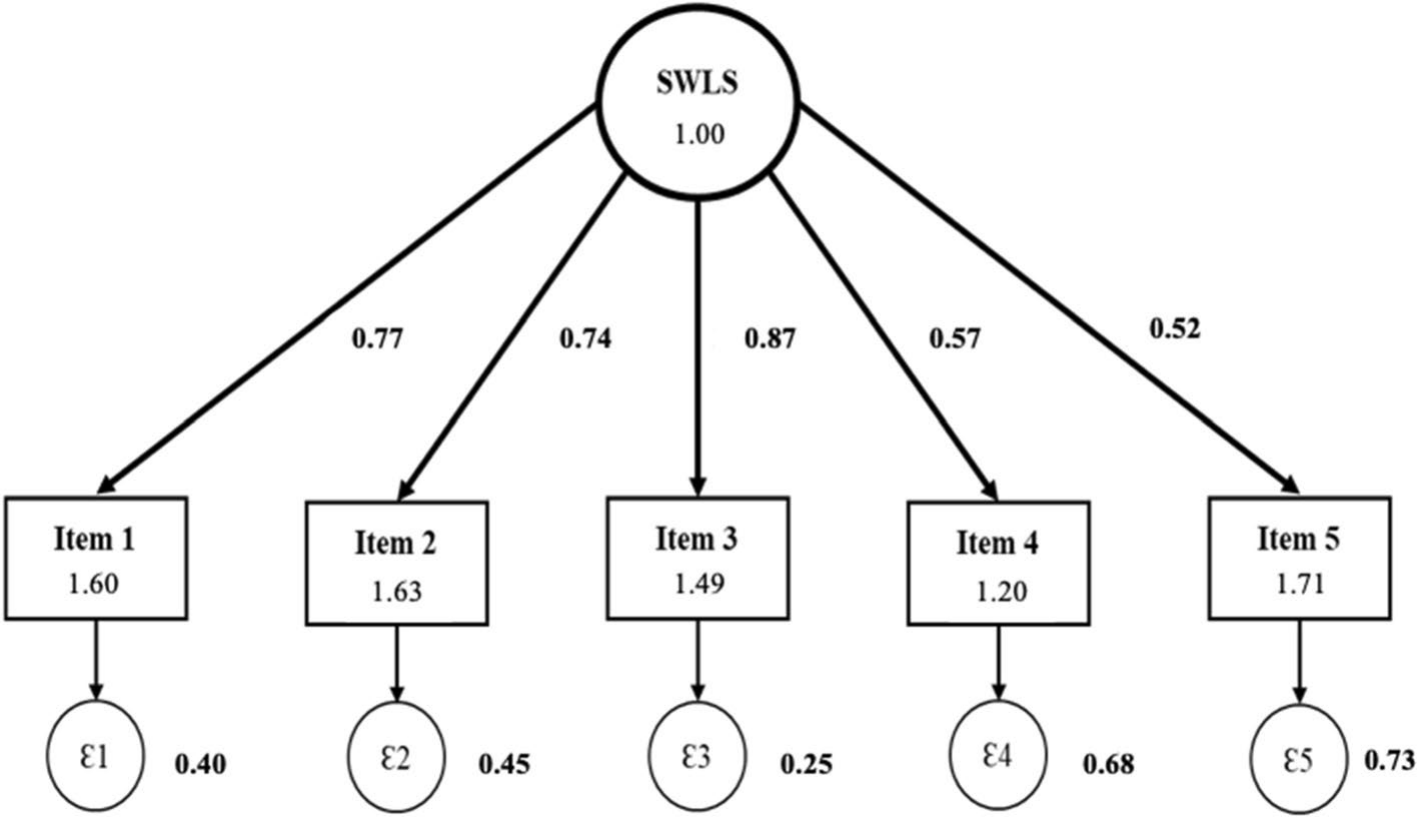
Validity analysis (confirmatory factor analysis) of the Satisfaction with Life Scale (SWLS)

Of the 342 invited students, 43.0% in Q1 and 65.8% in Q2 refused to participate. Table 1 shows the characterization of the sample in the two questionnaire applications. We observed greater participation among female (biology) students (68.7% in Q1 and 72.7% in Q2) and young people aged 21–25 years (44.6% in Q1 and 45.7% in Q2), but with no differential bias among demographic and academic distribution. Table 2 shows the reliability analysis (temporal stability and internal consistency) of the SWLS. All SWLS items showed acceptable reliability, with Spearman’s correlation coefficient ranging from 0.42 (item 4) to 0.73 (item 3). Cronbach’s alpha coefficient for SWLS items ranged from 0.72 to 0.83. Table 3 and Fig. 1 show the construct validity based on the internal structure (exploratory and confirmatory factor analysis) of the SWLS. Before the exploratory factor analysis, we observed that our data were factorable (KMO = 0.823; Bartlett’s test, *p* < 0.001). After that, we identified only one-factor structure, with an explained variance of 59.0%, and all items showed low communality values (up to ~ 0.6) (Table 3). In the confirmatory analysis, we identified a model adjusted for one factor (observed in the exploratory analysis), with the following parameters: *X*^2^/df of 6.53 (*p* = 0.258), TLI of 0.991, CFI of 0.996, RMSEA of 0.040, and SRMR of 0.026. Figure 1 graphically demonstrates the confirmed structure of the SWLS online format for university students.

**Table 1:** Sensitivity analysis and distribution of the sample in terms of demographic and academic variables of the reliability and validity study

**Variables**		**Q1% (*n* = 195)**^**a**^	**Q2% (*n* = 117)**^**b**^	***p*-value**^c^
Biological sex	Male	31.3	27.4	0.36
	Female	68.7	72.6	
Age	Up to 20 years	23.6	26.7	0.63
	21 to 25 years	44.6	45.7	
	26 to 30 years	18.5	14.7	
	31 to 35 years	7.2	5.2	
	36 years or more	6.2	7.8	
Academic course	Nutrition	8.8	6.0	0.17
	Law	14.1	11.1	
	Physical education	22.3	24.8	
	Nursing	11.1	12.0	
	Aesthetics and cosmetics	7.6	1.7	
	Physical therapy	16.1	18.8	
	Psychology	15.0	21.4	
	Social work	3.2	1.7	
	Administration	1.8	0.9	
Academic shift	Morning	20.1	20.5	0.92
	Afternoon	0.52	0.9	
	Evening	61.3	62.4	
	Full	18.0	16.2	

Significant values are in bold (*p* < 0.05). *Q1*, questionnaire first application; *Q2*, questionnaire first application

^a^Validity sample

^b^Reliability sample

^c^Chi-square goodness-of-fit test

**Table 2:** Reliability analysis of the Satisfaction with Life Scale (SWLS)

Items	Q1	Q2	Rho	Alpha
Item 1	2.0 (1.0–3.0)	2.0 (1.0–3.0)	**0.66**	0.74
Item 2	2.0 (1.0–4.0)	2.0 (1.0–3.0)	**0.69**	0.76
Item 3	2.0 (1.0–3.0)	2.0 (1.0–3.0)	**0.73**	0.72
Item 4	1.0 (1.0–2.0)	1.0 (1.0–2.0)	**0.42**	0.80
Item 5	3.0 (2.0–5.0)	3.0 (2.0–5.0)	**0.63**	0.83

Values are median (25th–75th percentile). *Alpha* Cronbach’s alpha coefficient, *Q1* Questionnaire first application, *Q2* Questionnaire second application; *rho*, Spearman correlation coefficient. Significant values are in bold (*p* < 0.05)

**Table 3:** Validity analysis (exploratory factor analysis) of the Satisfaction with Life Scale (SWLS)

Items	Factor 1	Uniqueness	Communality (1-uniqueness) %
Item 1	0.832	0.308	69.2
Item 2	0.798	0.364	63.6
Item 3	0.873	0.239	76.1
Item 4	0.675	0.545	45.5
Item 5	0.623	0.612	38.8
**Eigenvalue**	2.93		
**Explained variance**^a^	0.59 or 59.0%		

^a^Based on only one factor identified by using eigenvalue greater than one rule (Kaiser’s rule)

## Discussion

The main finding of our study was that the SWLS, in the online format, showed reliability and validity in identifying satisfaction with life among university students from a low-income region, an important risk factor for physical (Rogowska et al., 2021) and mental (Jensen et al., 2021) health in this population. Our findings suggest that the SWLS is a feasible tool with low economic and logistical costs and the ability to identify life satisfaction in conditions of limited personal access to students. The scale can be a useful alternative for research in a pandemic context, such as that experienced in recent years with COVID-19 (De Man et al., 2021).

We observed an increasing rejection/refusal rate from face-to-face recruitment (and response to Q1) to subsequent contacts via social network messages (and responses to Q2) in university students from a low-income region, but there was no differential bias between the two applications. A multicenter methodological study also observed a high prevalence of rejection/refusal in the recruitment phase in studies conducted in Latin America (Carvalho et al., 2018), with the proportion of rejection being higher at lower socio-economic levels or in lower income regions. Observational studies addressing European (Cena et al., 2021) and Brazilian (Barbosa et al., 2016) university students showed similar patterns. Regarding rejection/refusal during psychometric assessment (response to Q1 and Q2), our findings were in line with the literature if we consider that we excluded students who did not complete the SWLS. A comprehensive systematic review evidenced that the majority of the studies addressing scale development lost more than 50% of the initial item pool during the validation process (Morgado et al., 2017). Additionally, recent studies indicate that educational and socio-economic conditions may be a limiting factor for access to and adherence to online research in a pandemic context (De Man et al., 2021). Thus, we recommend that future studies in university students from low-income regions, with applications of online tools (including SWLS), conduct combined recruitment and participation approaches (e.g., adopt snowball sampling, allow participation via telephone of a relative or colleague, simultaneous reminders via social networks, SMS, and face-to-face, among others) (Hensen et al., 2021).

According to our results, the online SWLS showed acceptable reliability (temporal stability and internal consistency) among low-income university students. The literature evidenced internal consistency and test–retest stability as the most commonly used technique (Morgado et al., 2017) and a mandatory step in scale development and validation (Boateng et al., 2018). In this line, our findings corroborate a previous online study including European and South American university students (Rogowska et al., 2021), as well as Brazilian adults (Silva et al., 2018, 2021). The scientists identified acceptable internal consistency, with Cronbach’s alpha greater than 0.7, even in online versions in these populations. We attribute the reliability of the instrument to two main factors: (i) the use of short and simple statements (up to 13 words, item 5) and (ii) the fact that the statements referred to general aspects or conditions of life, conferring greater stability of perception for a short time interval (2 weeks).

Consistent with our findings, the literature indicates that the SWLS is a valid tool to identify latent satisfaction with life in adults (Hinz et al., 2018; López-Ortega et al., 2016; Rogowska et al., 2021; Silva et al., 2018, 2021). In this sense, previous studies conducted in Brazilian adults reported a one-factor structure solution for SWLS via exploratory (factor loadings > 0.60) (Silva et al., 2018) and confirmatory factor analysis (TLI ≥ 0.962; CFI ≥ 0.981; RMSEA ≤ 0.026; SRMR ≤ 0.040, for example) (Silva et al., 2018, 2021), reproducing the original SWLS structure solution (Diener et al., 1985). These previous studies supported our study hypothesis in which the SWLS theoretical factor structure extracted from a previous model was tested in a new sample of university students from a low-income region. Our results from the confirmatory factor analysis corroborated the one-factor structure solution for the online SWLS. A potential explanation for the consistent structural validity of the SWLS can be attributed to its invariance (which was not tested in the current study) (Arrindell et al., 2022; Silva et al., 2021), the ability of the tool to operationalize the concept of satisfaction with life in the same way with different samples (and consequently, populations) (Arrindell et al., 2022). Additionally, considering that the SWLS is a short and easy-to-fill tool (Hinz et al., 2018; Silva et al., 2021) and maintains its psychometric capacity in the online format, we recommend the scale for the tracking of life satisfaction in epidemiological studies. From our point of view, the SWLS meets conditions to be an applicable tool in the study of this outcome in a pandemic context (De Man et al., 2021).

Our study has some limitations. Although our sample was robust in size and diverse for age and sex, similar to the literature (Barbosa et al., 2016; Cena et al., 2021), the results of this study are restricted to psychometric findings. Additionally, we had a high proportion of rejections/refusals. In post hoc analysis, the power of the sample (lowest correlation observed = 0.42; *n* = 117) was 99.0% (*β* = 0.01), surpassing the base power of 90.0% in the sample estimate for reliability assessment, as well as our sample size was greater than the rule of thumb, which is a minimum of 10 participants for each item in the scale (in our case, 39 participants by each item) for validity assessment (Martınez-Gonzalez et al., 2014; Morgado et al., 2017). Furthermore, the research site was selected by convenience, and the sample was randomly selected. Since representative methodological studies are not feasible (Moreno et al., 2008), the sociodemographic, economic, and academic characteristics of the chosen institution were sufficient to provide the diversity of university students from low-income regions. Another factor that can be added is the level of education of the participants, who may have a better understanding of the questions and consequently provide more accurate answers, different from non-university adults from low-income regions. The SWLS is a subjective tool and is susceptible to measurement errors inherent to tools with these characteristics, such as memory bias and social desire (Szklo & Nieto, 2014).

## Conclusion

The Satisfaction with Life Scale, in the online format, is a reliable and valid tool for university students in a low-income context. The scale is a viable option for measuring life satisfaction when conditions do not permit face-to-face participation (e.g., isolation during a pandemic).

## Data Availability

The datasets used and/or analyzed during the current study are available from the corresponding author on reasonable request.

## Abbreviations

24 h-MESYN: 24-Hour Movement Behavior and Metabolic Syndrome
CFI: Comparative Fit Index
DEBQ: Dutch Eating Behavior Questionnaire
IPAQ: International Physical Activity Questionnaire
KMO: Kaiser–Meyer–Olkin test
*N*: Population
*n*: Sample
*p*: *p*-Value
PSQI: Pittsburgh Sleep Quality Index
PSS: Perceived Stress Scale
Q1: Questionnaire first application
Q2: Questionnaire second application
RMSEA: Root mean square error of approximation
SEM: Structural equation modeling
SRMR: Standardized root mean-squared residual
SWLS: Satisfaction with Life Scale
TLI: Tucker–Lewis Index
*X*^2^/df: Chi-square/degrees of freedom
*α*: Alpha
*β*: Beta
